# Gallstone Ileus in a Young Patient: A Clinical Case Report and Literature Review

**DOI:** 10.7759/cureus.33291

**Published:** 2023-01-03

**Authors:** Milton A Muñoz-Leija, Marion C Alemán-Jiménez, Alejandro Quiroga-Garza, Rodrigo E Elizondo-Omaña, Santos Guzmán-López

**Affiliations:** 1 General Surgery, Hospital General de Zona 6, Instituto Mexicano del Seguro Social, San Nicolas de los Garza, MEX; 2 Human Anatomy, Universidad Autónoma de Nuevo León, School of Medicine, Monterrey, MEX

**Keywords:** abdominal pain, bowel obstruction, surgical approach, gallstone ileus, gallbladder

## Abstract

Gallstone ileus is a rare presentation of gallbladder disease. It is mostly encountered in female and elderly patients. It occurs when a stone causes a fistula between the gallbladder and the intestinal lumen. More than half of the patients do not have a history of biliary disease. Surgical intervention is still considered the best treatment option; however, the best choice between one-stage and two-stage surgery is still unknown. We present a gallstone Ileus case in a patient with uncommon epidemiological characteristics: a 28-year-old male Hispanic patient without a gallbladder disease history.

## Introduction

Biliary or gallstone ileus is a late complication of gallstone disease in which a mechanical intestinal obstruction occurs due to the impaction of one or more gallstones within the lumen of the gastrointestinal tract [1]. This happens when the stone(s) causes a fistula between the gallbladder and the intestinal walls (most frequently with the duodenum), classified as a chronic fistula [2]. Depending on the size of the stone, it may become lodged in the ileocecal valve, although obstruction at the duodenum has also been reported [3]. It accounts for 1-4% of all bowel obstructions and occurs more frequently in elderly patients (≥65 years of age) and women (72-90% of all cases) [2,3]. Moreover, 50% of all patients presenting with gallstone ileus have a biliary disease history [3,4]. In this paper, we report the case of gallstone ileus in a patient with uncommon epidemiological characteristics.

## Case presentation

**Figure 1:**
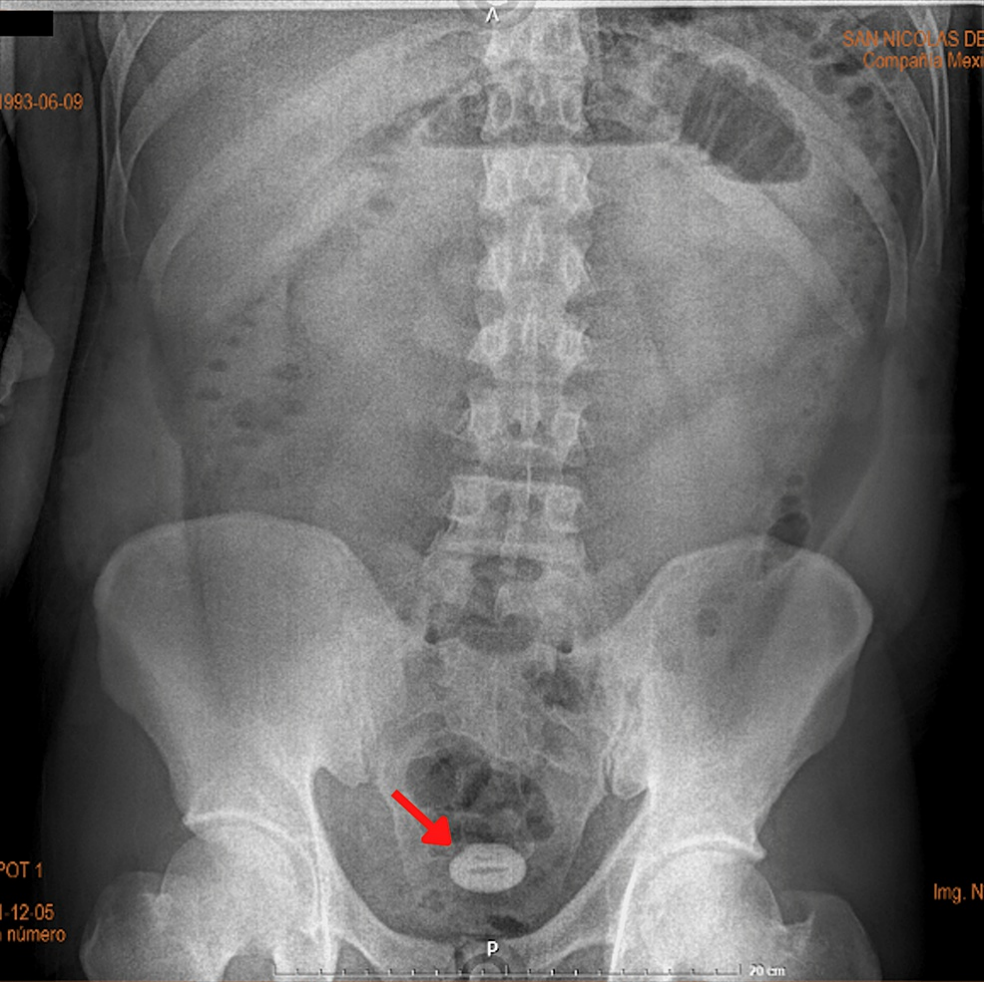
Abdominal X-ray showing the gallstone (the red arrow).

A 28-year-old Hispanic man presented to the emergency department with a three-day history of generalized abdominal pain and vomiting. The pain was abrupt and of colicky nature. He confirmed experiencing constipation, bloating, and not passing flatus for the last five days and denied a prior history of any comorbidities, abdominal surgery, cholelithiasis, or other of importance. The patient reported that he indulged in alcohol consumption for five consecutive days before symptoms started to appear and had two high-fat meals in the last two days. He denied any history of abdominal pain or biliary symptoms prior to this event. Upon assessment, the patient was hemodynamically stable and afebrile. Physical examination revealed a soft abdomen, mild mesogastric pain, and hypogastric tenderness. Initial laboratory workup was significant for leukocytosis (white blood cell count, 21.4 × 10⁹/L), neutrophilia (89.9%), a hemoglobin level of 18.3 g/dL, and amylase of 106 u/L; the rest of the parameters were unremarkable. A plain abdominal X-ray demonstrated a radio-opaque 3 cm × 3 cm density located in the hypogastric zone, and a dilated loop of the small bowel (Figure 1).

**Figure 2:**
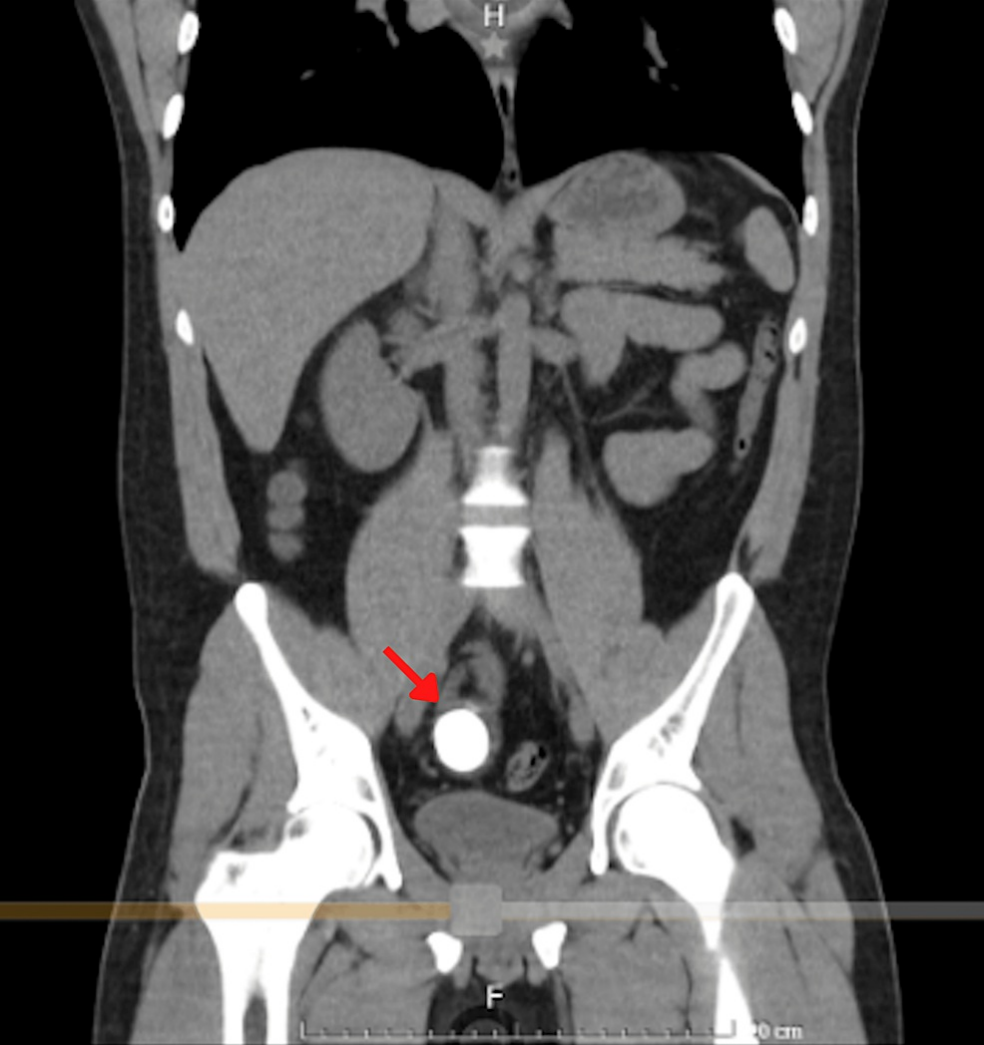
Coronal view of abdominal computed tomography revealing the gallstone (the red arrow) in the distal ileum.

**Figure 3:**
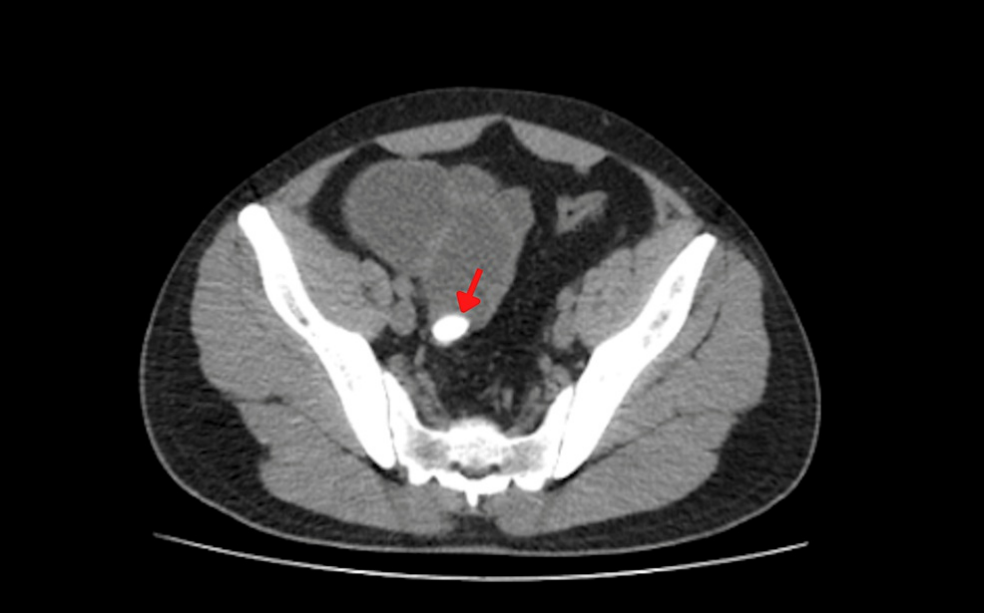
Axial view of abdominal computed tomography showing the gallstone in the distal ileum (the red arrow).

A plain abdominal computed tomography confirmed small bowel obstruction and revealed an abrupt transition point corresponding to a radio-opaque calculus in the ileum. No stones were observed in the gallbladder (Figure 2 and Figure 3).

**Figure 4:**
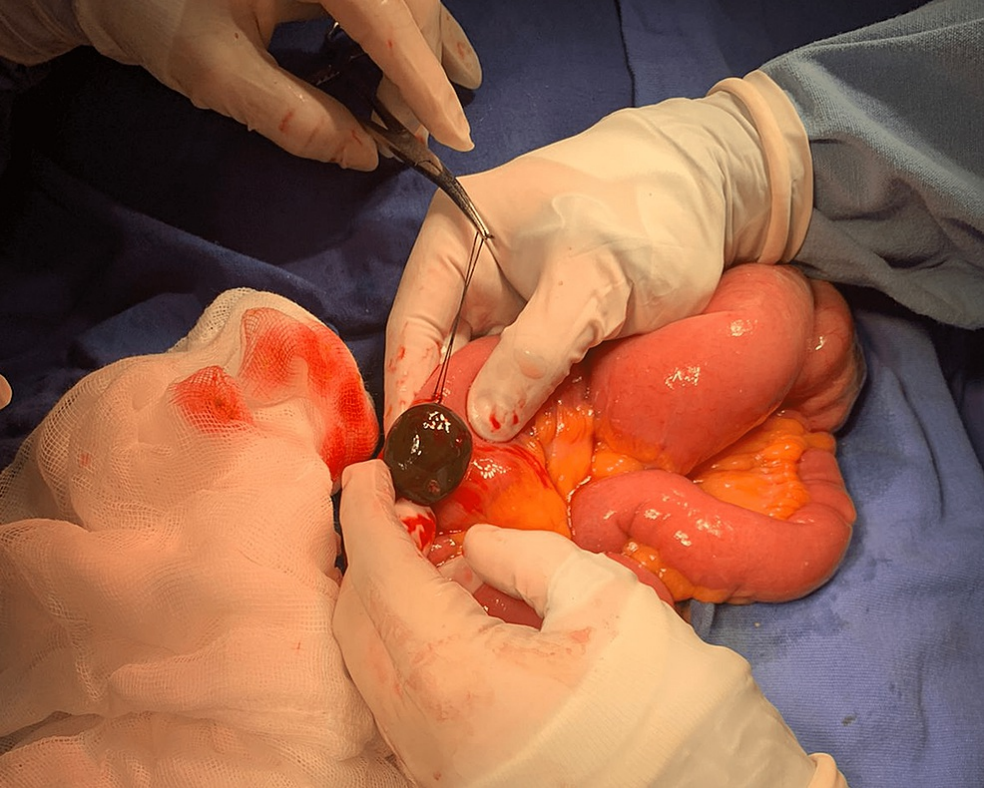
Gallstone impaction in the distal ileum.

**Figure 5:**
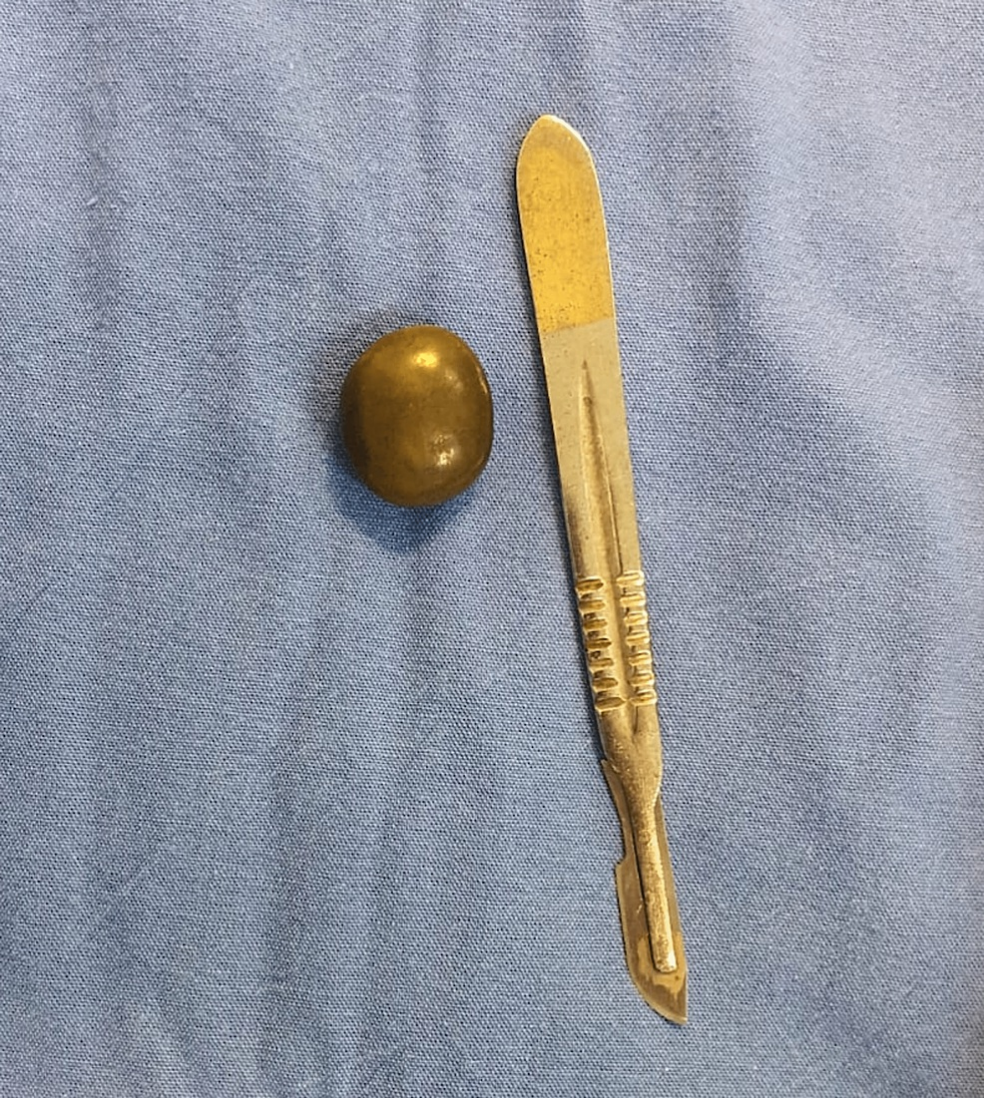
Removed gallstone

Resuscitative measures were taken. Dehydration was managed using crystalloid fluids, and decompression of the gastrointestinal tract was done using a nasogastric tube. Analgesic and prophylactic antibiotics with third-generation cephalosporin were administered. The patient was prepared for exploratory laparotomy. Intraoperative findings confirmed a large gallstone sized 30 cm at the ileocecal valve obstructing the lumen of the small bowel. A longitudinal 5 cm enterotomy was performed and a 4 × 3 × 3 cm gallstone was removed (Figure 4 and Figure 5).

No segmental bowel resection was required, and enterotomy was closed transversely. The post-operative period was uneventful. The patient had adequate recovery and was discharged two days after the procedure. Follow-ups at 14 days and six months after the procedure were also uneventful.

## Discussion

The term gallstone ileus is a misnomer as this condition is a mechanical obstruction of the gut and is not a true ileus. Dr. Erasmus Bartholin, a Danish physician, and mathematician first described gallstone ileus following an autopsy examination in 1654 [5]. Gallstone ileus commonly occurs in women and elderly (over 60 years old) populations. Other risk factors include a history of cholelithiasis, large stones (larger than 2 cm), episodes of acute cholecystitis, and those of Caucasian ancestry [3,6]. Our patient’s characteristics were not common for this pathology (young, male, thin, without a history of symptoms, risk factors, or diagnosed gallbladder disease). However, only 50% of the patients have a previous history of gallbladder disease [7]. To the best of our knowledge, this is the second young patient reported with gallstone ileus. The youngest patient was a 13-year-old Japanese patient, which was reported in a study with a 112-patient cohort published in 1980 [8-14] (Table 1).

**Table 1:** Youngest gallstone ileus cases reported in the literature. The table shows the youngest gallstone ileus cases reported in the literature, specifying relevant epidemiological characteristics such as sex and age. The surgical procedure carried out in each patient has also been shown.

Author, Year	Year	Country	Sex	Age	Surgery
Kasahara et al. [8]	1980	Japan	Male	13	Unknown
Micheletto et al. [9]	2013	Italy	Female	30	Enterolithotomy + retrograde cholecystectomy with transection and closure of the proximal end of the excluded jejunum.
Gupta et al. [10]	2010	India	Female	33	Exploratory laparotomy + gallstone removal through the perforation site + resection anastomosis
Morosin et al. [11]	2020	Australia	Male	34	Exploratory laparotomy + enterolithotomy
Teelucksingh et al. [12]	2018	Trinity & Tobago	Female	37	Exploratory laparotomy + enterolithotomy
Cruz-Santiago et al. [13]	2017	Mexico	Female	38	Exploratory laparotomy + enterolithotomy
Gachabayov et al. [14]	2016	Russia	Female	42	Exploratory laparotomy + enterolithotomy

The optimal management of acute gallstone ileus is contentious and can be divided into three subgroups: enterolithotomy alone, a one-stage procedure of enterolithotomy, cholecystectomy, and fistula closure, and a two-stage procedure of enterolithotomy with an interval cholecystectomy and fistula closure [3,4]. Simple enterolithotomy is both safe and effective and is associated with better outcomes than more invasive techniques. This procedure is suggested to be considered for low-operative-risk patients [15,16]. The laparoscopic versus conventional approach has not been studied. Reisner and Cohen [17] demonstrated that the one-stage procedure had a higher mortality rate compared with simple enterolithotomy. In the simple enterolithotomy group, 15% of patients had remaining biliary symptoms, of which only 10% required further surgeries for symptomatic relief. The recurrence of gallstone ileus was <5% in the same group. In this case, enterolithotomy was chosen as the procedure, and the patient had adequate evolution. According to the literature, more than half of cases present spontaneous closure of the fistula [4]; thus, it was decided not to address the fistula in the patient. Simple enterolithotomy can be considered an option in patients without comorbidities and young age. Surgeons should base their decision on their clinical judgment, expertise, and resources available. 

## Conclusions

Gallstone ileus is an unusual complication of cholelithiasis and most commonly occur in female patients of advanced age with previous history of gallbladder illness. However, in recent years, some reports provided opposite data. Surgical intervention is still considered the best treatment option; however, the choice among simple enterolithotomy, one-stage surgery, and two-stage surgery is still contentious. Surgeons must decide the best treatment for each patient according to their own expertise and available resources. Further research must be done to evaluate the best surgical procedure.
